# TAZ sensitizes EGFR wild-type non-small-cell lung cancer to gefitinib by promoting amphiregulin transcription

**DOI:** 10.1038/s41419-019-1519-z

**Published:** 2019-03-25

**Authors:** Weiwei Yuan, Wei Xu, Yan Li, Wei Jiang, Yue Li, Qiqing Huang, Bo Chen, Shuangshuang Wu, Yu Wang, Weiwei Song, Weihong Zhao, Jianqing Wu

**Affiliations:** 1 Jiangsu Provincial Key Laboratory of Geriatrics, Department of Geriatrics, The First Affiliated Hospital of Nanjing Medical University, Nanjing, China; 2 Department of Thoracic and Cardiovascular Surgery, The First Affiliated Hospital of Nanjing Medical University, Nanjing, China; 3 Nanjing Lishui People’s Hospital, Zhongda Hospital Lishui Branch, Southeast University, Nanjing, China

## Abstract

Comparatively less toxic and more tolerated, epidermal growth factor receptor-tyrosine kinase inhibitors (EGFR-TKIs) are recommendable for advanced non-small-cell lung cancer (NSCLC) patients with EGFR-sensitive mutations. Some EGFR wild-type patients with specific biomarkers also show a response to the drug. TAZ is an oncogene closely associated with the therapeutic effect of EGFR-TKIs. However, this association remains to be clarified. This study aimed to clarify the mechanism through which TAZ sensitizes EGFR wild-type NSCLC to gefitinib. We used CCK-8 assays and in vivo experiments to investigate the influence of TAZ on gefitinib in EGFR wild-type NSCLC. To further validate the tumorigenic role of TAZ, we performed Human umbilical vein endothelial cell (HUVEC) tube formation and migration assays. Luciferase reporter assays, quantitative real-time PCR (qPCR), immunoblotting and Chromatin immunoprecipitation collaborated with qPCR illuminated the mechanism through which TAZ caused those phenotypes. The results showed TAZ promoted the angiogenesis of NSCLC cell lines and improved gefitinib sensitivity in EGFR wild-type NSCLC in vitro and in vivo. Luciferase reporter assays and ChIP-qPCR experiments showed TAZ upregulated AREG by promoting its transcription. EGFR signaling pathway was activated as TAZ was highly expressed. Rescue experiments were conducted to confirm the indispensable role of AREG in tumorigenesis and gefitinib sensitivity regulated by TAZ. Our study concluded that TAZ sensitized EGFR wild-type NSCLC to gefitinib through promoting amphiregulin transcription.

## Introduction

Of all malignancies, lung cancer has the highest morbidity and mortality worldwide^1^. About 85% of lung cancer cases fall victim to non-small-cell lung cancer (NSCLC)^2^. Platinum-based chemotherapy combined with thoracic radiation serves as the standard treatment for advanced NSCLC patients ineligible for surgery^3^. However, its therapeutic efficacy is limited, as evidenced by the low 5-year survival rate^4^.

Compared with docetaxel or pemetrexed, epidermal growth factor receptor-tyrosine kinase inhibitors (EGFR-TKIs) present higher tolerability and less toxicity in advanced NSCLC patients with EGFR-sensitive mutations^5^. EGFR-TKI can prolong the progression-free survival of selected patients^6^. Gefitinib is the first targeted drug approved to treat selected NSCLC patients. Gefitinib reversibly binds to EGFR tyrosine kinase domain and competitively inhibits ATP binding and downstream phosphorylation, thus suppressing tumor growth mediated by EGFR signaling pathway^7^. EGFR-TKIs were proven to be effective as first-line drugs in patients with EGFR-sensitive mutations^3^. However, several studies revealed that patients with wild-type EGFR also benefit from it^8,9^, the mechanism of which is not yet clear.

First discovered in *Drosophila*, transcriptional co-activator with the PDZ-binding motif (TAZ) plays a key role in the Hippo pathway^10^. Clinical studies have found the protein expression of TAZ is closely related to malignancies, including breast cancer^11^, glioma^12^, colorectal cancer^13^, and lung cancer^14^. Japanese scientists found that gefitinib-sensitive genes correlated with TAZ expression, and that amphiregulin (AREG), a ligand of EGFR, may be the downstream target of TAZ by microarray analysis^15^. Basic and clinical studies also showed that AREG may be used as a biomarker to select EGFR wild-type NSCLC patients who benefit from gefitinib treatment^16,17^.

Based on the above findings, we speculated that in EGFR wild-type NSCLC, TAZ might upregulate the expression of AREG, activate the EGFR signaling pathway and sensitize EGFR wild-type NSCLC to gefitinib.

## Materials and methods

### Cell culture and gefitinib treatment

All human NSCLC cells (A549, H460, H358, and H1299) and normal bronchial epithelial cells (16HBE) were purchased from the Chinese Academy of Sciences (Shanghai, China) and cultured in DMEM supplemented with 10% fetal bovine serum (ScienCell, Carlsbad, CA, USA) and 1% penicillin–streptomycin (Gibco, Carlsbad, CA, USA) in a humidified atmosphere containing 5% CO_2_. The cells were challenged with gefitinib (Selleck, Houston, TX, USA) at different concentrations (Fig. 4).

### Plasmids and transfection

Short-hairpin RNAs (shRNAs) targeting TAZ (shTAZ), TEAD (shTEAD), and AREG (shAREG) and human gene expression plasmids pEX2-TAZ and pEX3-AREG were obtained from GenePharma (Shanghai, China). The target sequences were as follows: shTAZ: AGGTACTTCCTCAATCACA; shAREG: CACTGCCAAGTCATAGCCATAC; shTEAD: ATGATCAACTTCATCCACAAG. The cells were transfected using Lipofectamine 2000 (Invitrogen, Carlsbad, CA, USA). In specific experiments, the plasmids were transfected alone or in combination. The cells showing stable expression were selected with G418.

### Western blotting and antibodies

Total protein was harvested after cell treatment, and the protein level was determined using the BCA protein assay kit (Thermo Scientific, Rockford, IL, USA). Then, 30 µg of lysate was separated via 10–15% SDS-PAGE (Beyotime, Jiangsu, China), and the protein was transferred onto a PVDF membrane. The membrane was incubated with primary antibodies at 4 °C overnight and HRP-conjugated IgG at room temperature for 2 h. Also used were antibodies specific to TAZ (1:1000, Cell Signaling Technology, Boston, MA, USA), EGFR (1:1000, CST, USA), phospho-EGFR (1:1000, CST, USA), AKT (1:1000, CST, USA), phospho-AKT (1:1000, CST, USA), ERK1/2 (1:1000, CST, USA), phospho-ERK1/2 (1:1000, CST, USA), AREG (1:614, R&D System, Minneapolis, MN, USA), PECAM-1 (1:1000, Affinity Biosciences, USA), PROX-1 (1:1000, abcam, Cambridge, MA, USA). An antibody specific to GAPDH (1:1000, Wanleibio, Shenyang, China) was used as an internal control.

### Reverse transcriptase-qPCR

Total RNA was extracted using TRIzol reagent (Invitrogen, San Diego, CA). Then, cDNA synthesis was performed with a Goldenstar^TM^ RT6 cDNA Synthesis Kit (TsingKe Biotech, Beijing, China), and qPCR with 2× T5 Fast qPCR Mix (TsingKe Biotech, Beijing, China). The experiment was carried out on a StepOnePlus^TM^ Real-Time PCR System (Applied Biosystems, Foster City, CA, USA). GAPDH was selected as an endogenous control. The relative expression level was calculated with the formula of 2^−ΔΔCT^. All of the experiments were performed for at least three times. The primer sequences: GAPDH-F: GGTGAAGGTCGGAGTCAACGGA; GAPDH-R: GAGGGATCTCGCTCCTGGAAGA; TAZ-F: AGTACCCTGAGCCAGCAGAA; TAZ-R: GATTCTCTGAAGCCGCAGTT; AREG-F: CGTGTCCCAGAGACCGAGTT; and AREG-R: AGGTCCAATCCAGCAGCATAATG.

### Cell viability assay

Cell Counting Kit-8 (CCK-8; Beyotime, Jiangsu, China) was applied to measure cell viability. The cells were seeded in 96-well plates (3000 cells/well). After 10 h of incubation for cell adherence, gefitinib at different concentrations was given. The cells were incubated for another 48 h. Then, 10 µl of CCK-8 reagent was added into each well, followed by 1.5 h of incubation at 37 °C. The reaction was evaluated according to the absorbance at 450 nm. All experiments were performed in six to eight duplicate wells, and all experiments were performed for at least three times.

### Chromatin immunoprecipitation (ChIP)

A total of 4 × 10^6^ cells were harvested and crosslinked by formaldehyde at a final concentration of 1%. After stopping crosslink with glycine, chromatin was sheared to 100–300 bp with sonication. The anti-TAZ antibody (CST, #4883) and protein G beads were applied to pull down the target protein. Then the protein was digested with proteinase K. DNA (binding to the interest protein) was harvested and purified. Immunoprecipitated DNA was analyzed by qPCR.

### Luciferase reporter assay

H460 cells were seeded in 24-well plates at a confluence of 70%. After 16 h, 100 ng of the firefly luciferase reporter plasmid (pGL4.23-AREG promoter-wt/pGL4.23-AREG promoter-mut) and 100 ng of pEX2-TAZ/pEX2 vector were added for co-transfection. In addition, 10 ng of the Renilla luciferase pRL-TK plasmid was used as the loading control. After 48 h, luciferase activities were detected with the Dual-Luciferase Reporter Assay System (Promega, Madison, WI, USA) in a GloMax 96 Microplate Luminometer (Promega, USA). Relative activity was assessed using the ratio of the firefly luciferase signal to the Renilla luciferase signal.

### HUVEC tube formation and migration assays

H460 and A549 cells were transfected, and when they reached a confluence of 80%, the media were substituted with serum-free DMEM to culture the cells. After 24 h, the supernatant was collected. For the tube formation assay, every 2 × 10^4^ HUVECs were seeded in a 96-well plate precoated with 50 µl of Matrigel (BD Biosciences, Franklin Lakes, NJ, USA). Different supernatants were used as conditioned media to maintain the HUVECs. Images were captured after 4 h of incubation, and the total branch length in each well was measured using ImageJ. For the HUVEC migration assay, 24-well hanging inserts with 8.0 µm pores were obtained from Millipore. The cells were resuspended in DMEM and counted. Four hundred microliters of DMEM containing 20,000 cells were placed in the upper chamber. Conditioned media was placed in the lower chamber. After a specific incubation, the cells were fixed with methanol and stained with crystal violet. The unmigrated cells in the upper chambers were wiped away with cotton swabs. The migrated cells in three random fields were counted under a light microscope. Each assay was repeated for at least three times.

### Tumor xenograft model

Four-week-old female BALB/c nude mice were purchased from the Department of Laboratory Animal Center of Nanjing Medical University. The use of all experimental animals complied with the protocols set by Nanjing Medical University (NJMU) Institutional Animal Care and Use Committee. A total of 1 × 10^6^ stably transfected cells were injected subcutaneously into the flanks of the nude mice. For the drug in vivo experiments, gefitinib (10 mg/kg/day) in 5% glucose was given orally. Tumor size was measured with Vernier calipers every 4 d for 28 d. The tumors were harvested for histopathologic examination. For immunohistochemistry (IHC), fixed samples were embedded in paraffin. Four-micrometer-thick sections were incubated with primary antibodies specific to TAZ, pEGFR, Ki67, and CD34 at 4 °C overnight. The slides were then incubated with MaxVision^TM^ 2 followed by the DAB chromogen and hematoxylin counterstain. The slides were checked in a blinded manner. Images of three randomly chosen fields on each slide were captured under a microscope.

### Statistical analysis

The data were expressed as the mean ± standard deviation (SD). The analysis was carried out using SPSS 20.0 (SPSS, Inc., Chicago, IL, USA). Independent *t*-test was used to evaluate between-group difference. *p* < 0.05 was considered statistically significant. *: *p* < 0.05; **: *p* < 0.01; ***: *p* < 0.001.

## Results

### TAZ promoted the angiogenesis in EGFR wild-type NSCLC cell lines

**Fig. 1:**
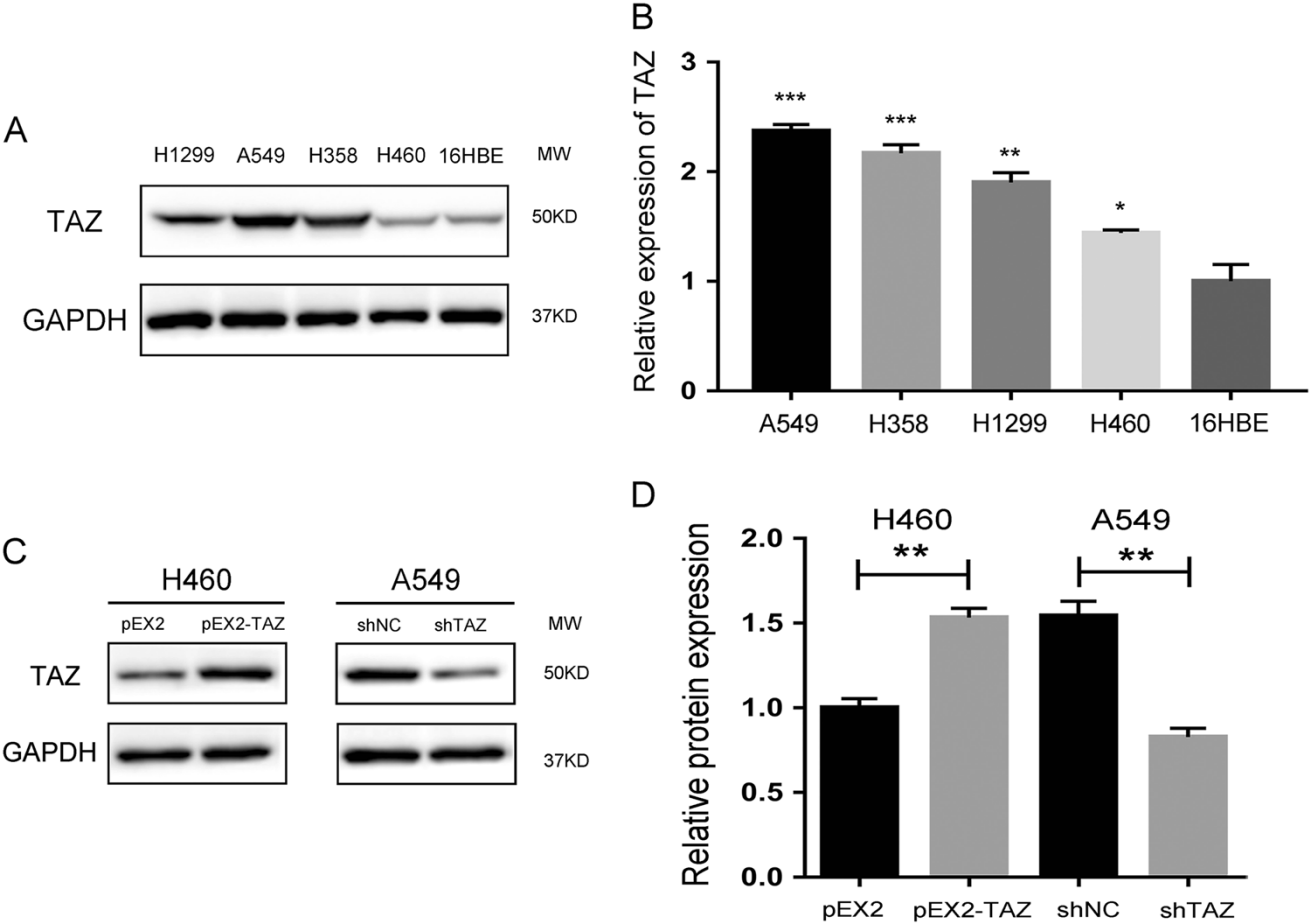
The expression of TAZ in NSCLC cell lines. **a**, **b** The protein expression level of TAZ in four EGFR wild-type NSCLC cell lines (H1299, A549, H358, and H460) and one normal human bronchial epithelial 16HBE cell line was detected by western blotting. **c** Efficiency of transfection was detected by western blotting. H460 cells were transfected with pEX2-NC or pEX2-TAZ, A549 cells were transfected with shNC or shTAZ. GAPDH was used as the loading control. Data were shown as mean ± SD. Each experiment was repeated for at least three times. *: *p* < 0.05; **: *p* < 0.01; ***: *p* < 0.001

According to the Cancer Cell Line Encyclopedia Database, we selected out four EGFR wild-type NSCLC cell lines (A549, H460, H1299, and H358) and one normal human bronchial epithelial cell line (16HBE) for experiments. The four NSCLC cell lines showed no EGFR mutation (data not shown) but expressed TAZ (Fig. 1a, b).The TAZ expression level was the highest in A549 cells and lowest in H460 cells. Therefore, A549 cells were used for TAZ knockdown and H460 cells for TAZ-overexpression experiment. The transfection efficiency was validated by western blotting (Fig. 1c, d).

**Fig. 2:**
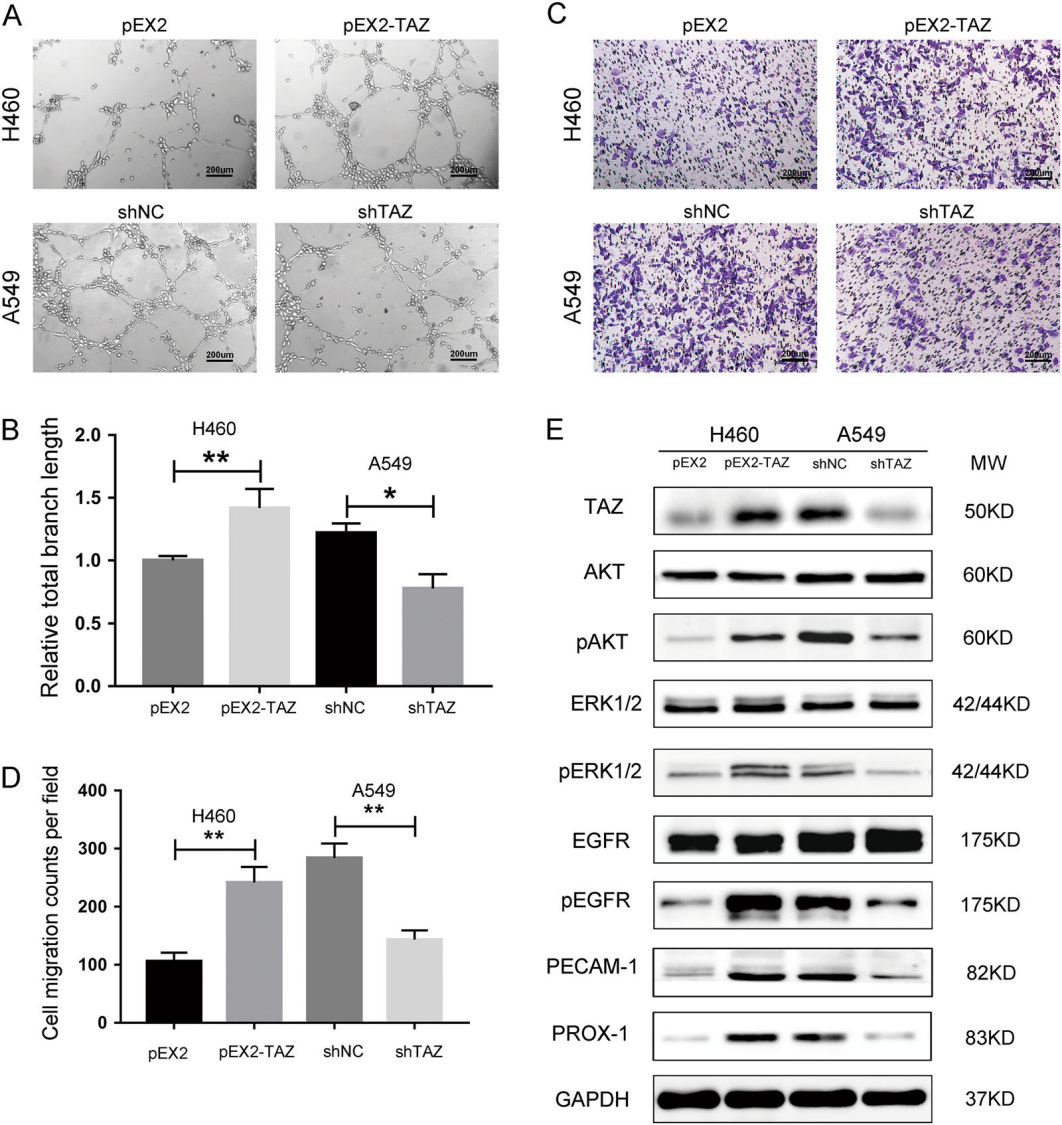
TAZ promoted the angiogenesis of NSCLC cells and activated EGFR signaling. **a**–**d** HUVEC cells were used to form tubes and transfer the chambers in the presence of conditional media. **e** Cell lysates from transfected H460 and A549 cells were probed with antibodies for TAZ, EGFR signaling and endothelial markers. GAPDH was used as the loading control. Data were shown as mean ± SD. Each experiment was repeated for at least three times. The scale bar is 200 µm. *: *p* < 0.05; **: *p* < 0.01

Compared to those in the control group, HUVECs cultivated in H460-derived medium exhibited a stronger tube-forming capacity that dramatically reduced when these cells further cultivated in A549-derived medium in the absence of TAZ (Fig. 2a, b). In addition, HUVEC migration was remarkably enhanced by highly expressed TAZ, and suppressed by lowly expressed TAZ (Fig. 2c, d). These results demonstrated TAZ can regulate tumor angiogenesis in EGFR wild-type NSCLC cell lines.

### TAZ-activated EGFR signaling pathway in EGFR wild-type NSCLC cell lines

High expression of TAZ induced high-level phospho-Akt, phospho-ERK1/2 and phospho-EGFR, and low expression of TAZ induced the opposite result. However, no remarkable difference was observed in the expression of total-Akt, total-ERK1/2 or total-EGFR (Fig. 2e). As shown in Fig. 2e, the expression of two endothelial markers (PECAM-1 and PROX-1) increased as TAZ was overexpressed. However, the expression of PECAM-1 and PROX-1 decreased in cells in the absence of TAZ. These results indicated TAZ-activated EGFR signaling pathway.

### TAZ promoted tumor growth of NSCLC in vivo

**Fig. 3:**
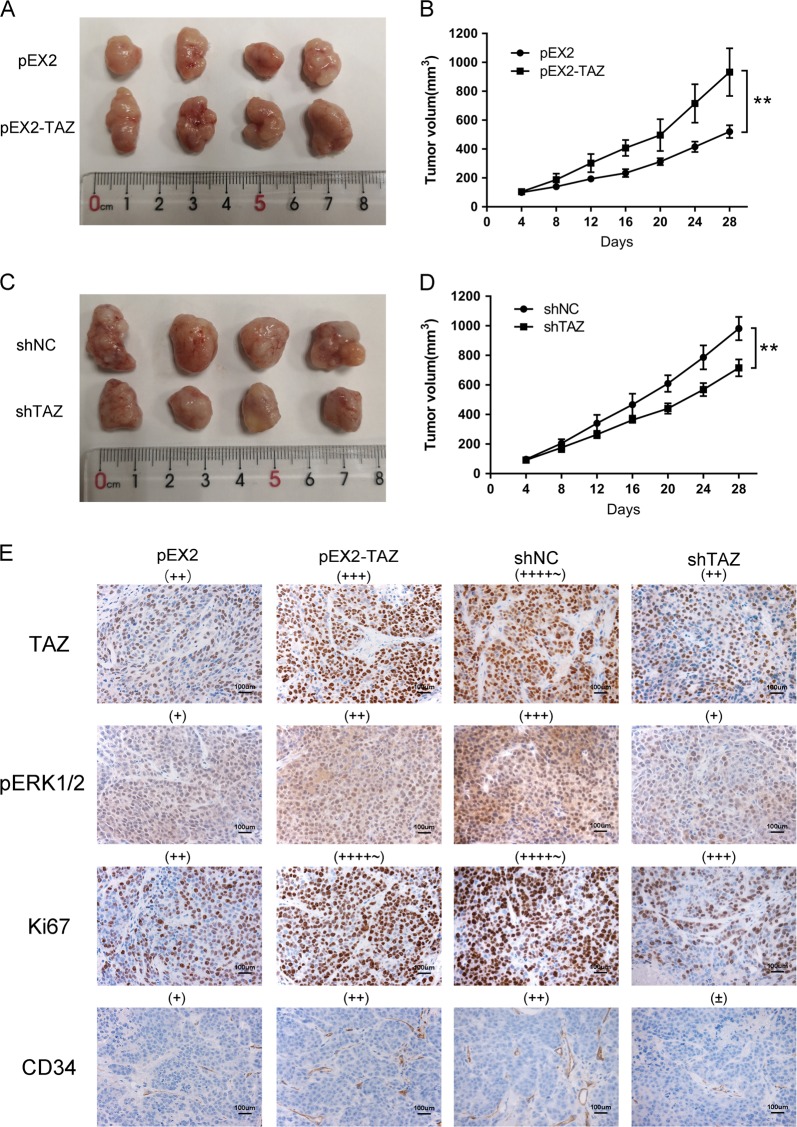
TAZ promoted tumor growth of NSCLC in vivo. **a**, **c** Photographs of subcutaneous xenografts derived from transfected H460 cells and A549 cells. **b**, **d** Growth curve of tumor volumes in different groups during 28 days. **e** Immunohistochemistry was applied to stain against TAZ, pERK1/2, Ki67, and CD34. Ki67 indicated the proliferative ability and CD34 the angiogenic ability. Data were shown as mean ± SD. Each experiment was repeated for at least three times. The scale bar is 100 µm. **: *p* < 0.01

To validate the oncogenic role of TAZ in vivo, we inoculated stably transfected H460 cells and A549 cells into nude mice. As shown in Fig. 3a, b, xenografts collected in the group of TAZ overexpression exhibited larger volume compared with those in the control group. Meanwhile, knockdown of TAZ reduced the growth of tumors (Fig. 3c, d). According to immunohistochemical analyses, the expression of TAZ was consistent with that in the mice treated as described above. The staining of phospho-ERK1/2 and Ki67 was enhanced in TAZ-overexpression group and weakened in TAZ low expression group. The distribution of CD34 revealed more obvious angiogenesis in TAZ-overexpression group (Fig. 3e). These findings indicated that TAZ promoted tumorigenesis in vivo.

### TAZ sensitized EGFR wild-type NSCLC to gefitinib

**Fig. 4:**
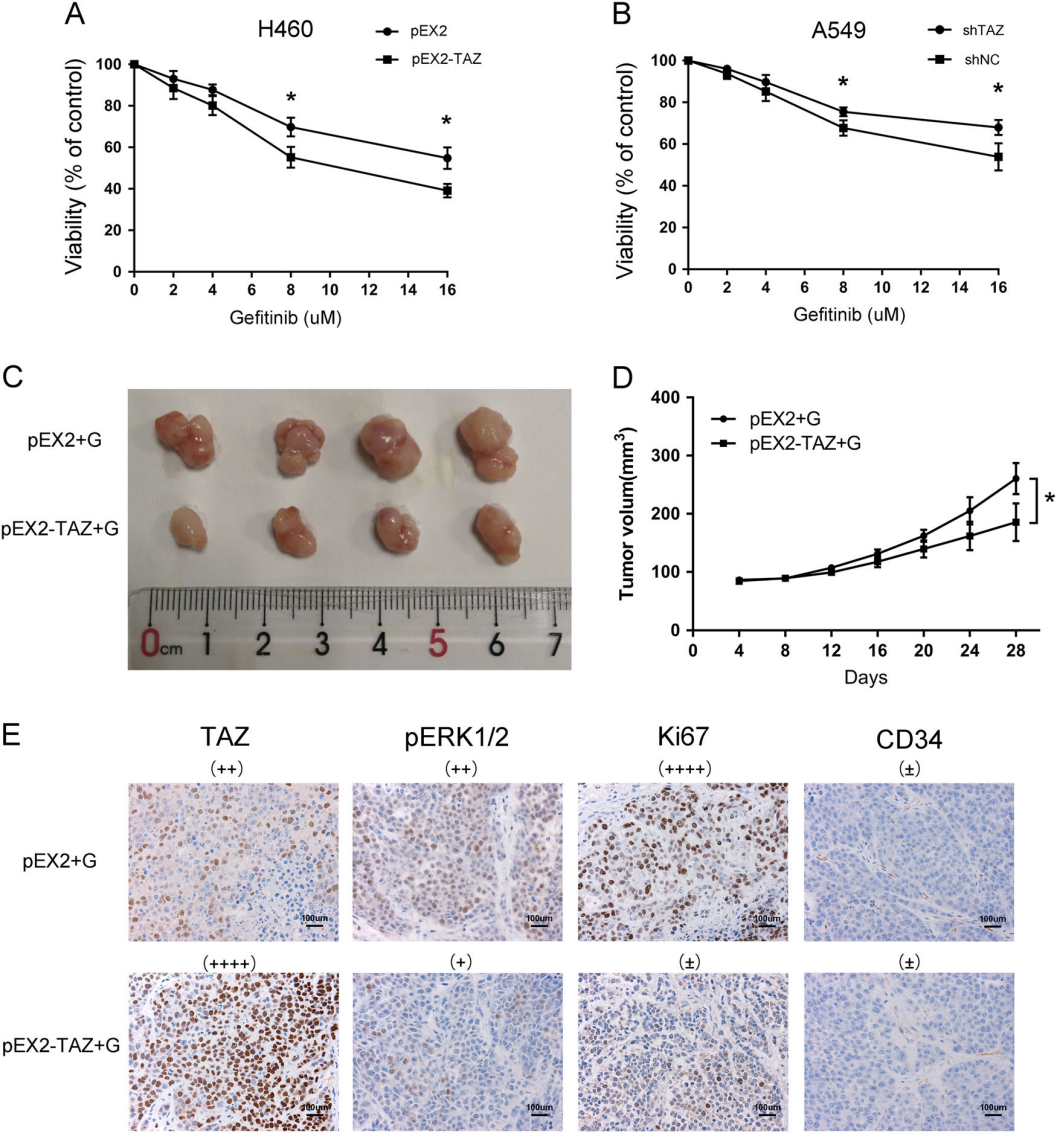
TAZ improved the sensitivity of EGFR wild-type NSCLC to gefitinib. **a**, **b** H460 and A549 cells were transfected and exposed to gefitinib at a concentration gradient for 48 h. The viability of cells was measured by a CCK-8 assay and calculated with the following formula: viability rate = OD (treated)/OD (untreated) × 100%. **c** Photographs of subcutaneous xenografts derived from transfected H460 cells subsequently treated with gefitinib. **d** Growth curve of tumor volumes during 28 days. **e** Immunohistochemistry was applied to stain against TAZ, pERK1/2, Ki67, and CD34. G represents gefitinib. Data were shown as mean ± SD. Each experiment was repeated for at least three times. The scale bar is 100 µm. *: *p* < 0.05

After the administration of gefitinib, the overexpression of TAZ weakened the viability of H460 cells, and the knockdown of TAZ strengthened the cell viability of A549 cells, compared with the control group (Fig. 4a, b). As shown in Fig. 4c, d, with the administration of gefitinib, the mice injected with H460 pEX2-TAZ cells developed markedly smaller tumors than those injected with H460 pEX2 cells. According to the results of immunohistochemistry, gefitinib inhibited the staining of pERK1/2 and Ki67 in TAZ overexpression group than in the control group. However, CD34 staining displayed no difference between the two groups. (Fig. 4e) Taken together, TAZ improved the sensitivity of EGFR wild-type NSCLC to gefitinib in vitro and in vivo.

### TAZ regulated AREG in EGFR wild-type NSCLC

**Fig. 5:**
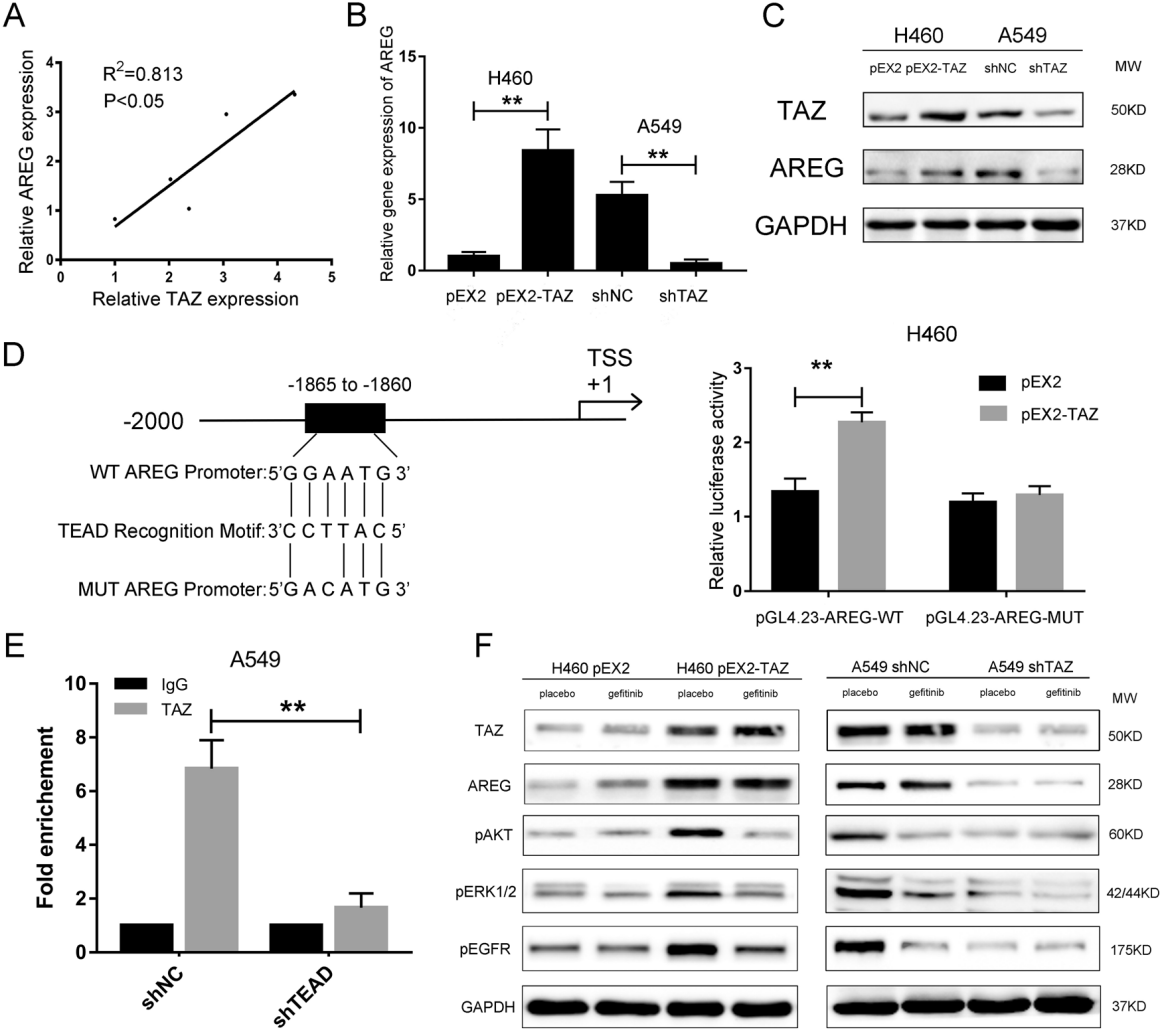
TAZ regulated EGFR signaling pathway via AREG. **a** Gene expression of TAZ and AREG was detected by qPCR and a two-tailed Pearson’s correlation analysis was performed. **b** Gene expression of AREG in transfected H460 and A549 cells was detected by qPCR. **c** Protein expression level of TAZ and AREG in transfected H460 and A549 cells was measured by western blotting. GAPDH was used as the loading control. **d** H460 cells were co-transfected with pGL4.23-AREG-WT + pEX2-TAZ; pGL4.23-AREG-WT + pEX2; pGL4.23-AREG-MUT + pEX2-TAZ; pGL4.23-AREG-MUT + pEX2. Relative luciferase activity was shown in the histogram. **e** Chromatin immunoprecipitation together with qPCR was performed in shTEAD condition. **f** Transfected cells were exposed to placebo (PBS) or gefitinib at the concentration of 8 µM. Cells were harvested after 3 h and the protein expression of TAZ, AREG, pAKT, pERK, and pEGFR were measured by western blotting. GAPDH was used as the loading control. Data were shown as mean ± SD. Each experiment was repeated for at least three times. **: *p* < 0.01

Based on the previous results of microarray analysis^15^, we analyzed the gene expression of AREG and TAZ in the five cell lines (A549, H460, H1299, H358, and 16HBE) using two-tailed Pearson’s correlation analysis. The results showed a positive association between TAZ and AREG expressions (Fig. 5a). To support this finding, we detected the expression of AREG in transfected A549 and H460 cells. The gene expression of AREG was significantly increased in TAZ-overexpressed H460 cells, and lowered by the knockdown of TAZ (Fig. 5b). In accordance with the qPCR results, western blotting results also suggested that the protein expression of AREG increased when TAZ was overexpressed in H460 cells and decreased when TAZ was silenced in A549 cells (Fig. 5c). These findings suggested AREG acted as a downstream target of TAZ.

### TAZ activated the transcription of AREG in EGFR wild-type NSCLC

TEAD, the major transcription factor binding to TAZ via the TEA domain, cannot function without a transcriptional co-activator, such as TAZ. That is to say, TAZ needs to pair with TEAD to transcribe^18^. To determine the potential mechanism through which TAZ regulates the expression of AREG, we consulted JASPAR database (http://jaspar.genereg.net/), finding that the upstream sequence of AREG promoter region matched to the TEAD recognition motif. This suggested TAZ might upregulate AREG through activating its transcription with the help of TEAD. To verify this assumption, we applied dual-luciferase reporter assays and ChIP-qPCR assays. H460 cells with low endogenous TAZ expression were co-transfected with pGL4.23-AREG-WT/pGL4.23-AREG-MUT and pEX2-TAZ /pEX2. The luciferase activity of pGL4.23-AREG-WT was distinctly enhanced after co-transfection with pEX2-TAZ compared to that co-transfected with the pEX2 vector. However, no distinct difference was found in the luciferase activity of pGL4.23-AREG-MUT (Fig. 5d). As shown in Fig. 5e, the sample in which TEAD was knocked down showed significantly lower fold enrichment of AREG, implying that TAZ had been recruited to AREG gene promoters via TEAD. Taken together, these findings suggested AREG served as a downstream target of TAZ via TEAD.

### TAZ-activated EGFR signaling pathway sensitized EGFR wild-type NSCLC cell lines to gefitinib

As AREG is a ligand of EGFR, we surmised TAZ may activate EGFR signaling pathway via AREG. As shown in Fig. 5f, the expression of AREG was changed by TAZ, regardless of whether gefitinib or placebo was added. The phosphorylation of Akt, ERK1/2 and EGFR was notably inhibited by gefitinib in TAZ-overexpressed H460 cells. Meanwhile, in A549 cells which transfected with negative control shRNA, the phosphorylation of Akt, ERK1/2 and EGFR was also inhibited by gefitinib compared with that in the placebo treated group. However, in H460 pEX2 cells and A549 shTAZ cells, no significant difference was detected between the two groups. These results indicated that TAZ enhanced the sensitivity of EGFR wild-type NSCLC cells to gefitinib by facilitating the phosphorylation of Akt, ERK1/2 and EGFR.

### TAZ enhanced the tumorigenic ability and drug sensitivity of EGFR wild-type NSCLC cells by upregulating AREG

**Fig. 6:**
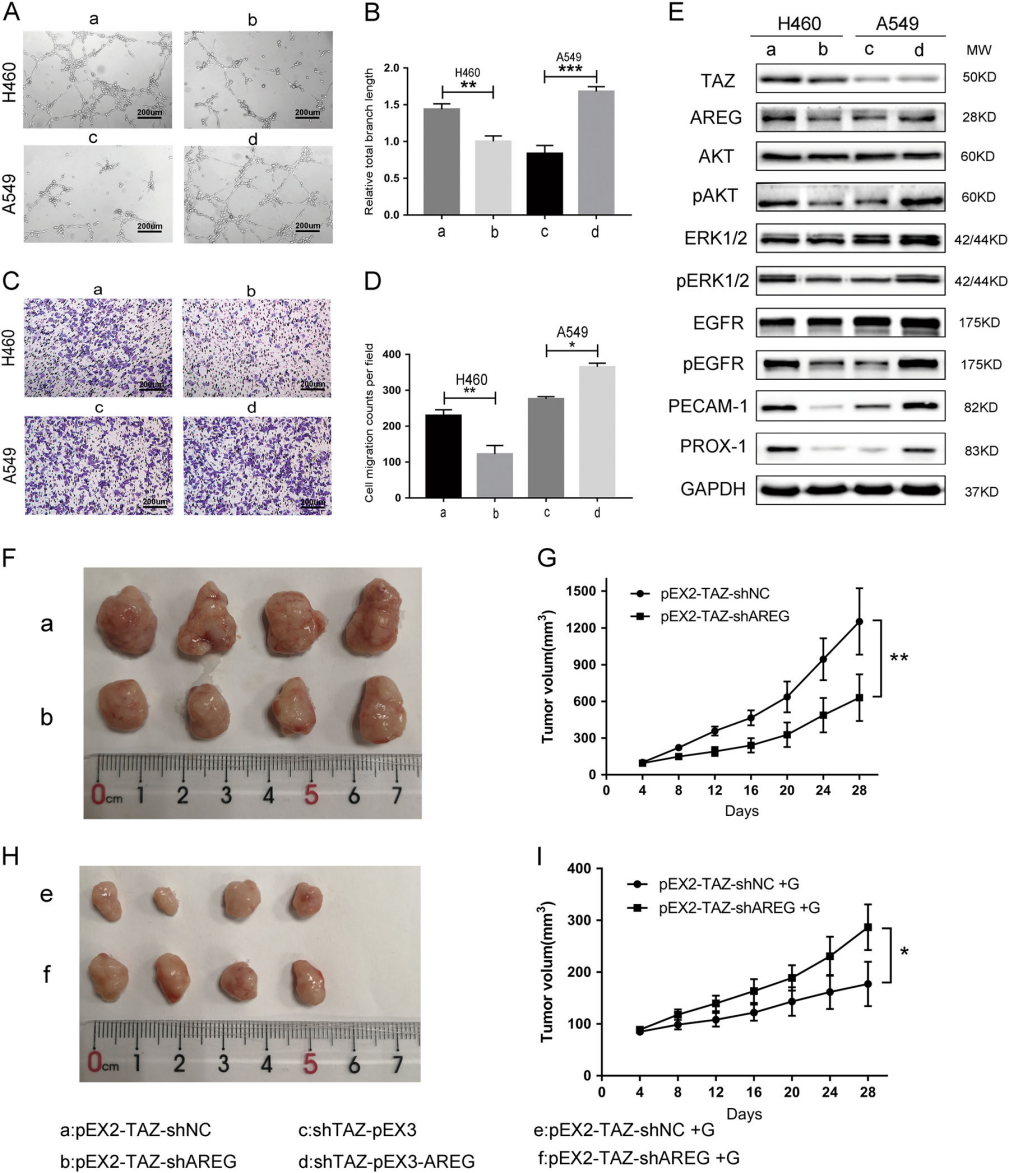
TAZ promoted tumor growth and affected gefitinib sensitivity by AREG. H460 cells were co-transfected with pEX2-TAZ and shAREG or shNC. A549 cells were co-transfected with shTAZ and pEX3-AREG or pEX3. Angiogenesis ability was demonstrated by **a**–**d**, HUVEC tube formation, and migration assays. **e** Protein expression levels of TAZ, AREG, and EGFR signaling and endothelial markers were detected by western blot. GAPDH was used as the loading control. **f**, **h** Photographs of subcutaneous xenografts derived from H460 cells co-transfected with pEX2-TAZ and shNC or shAREG and the relevant gefitinib-treated groups. **g**, **i** Growth curve of tumor volumes during 28 days. G represents gefitinib. Data were shown as mean ± SD. Each experiment was repeated for at least three times. The scale bar is 200 µm. *: *p* < 0.05; **: *p* < 0.01; ***: *p* < 0.001

To illustrate the pivotal role of AREG in TAZ-mediated phenotypes, we conducted rescue assays. As shown in Fig. 6a, the knockdown of AREG inhibited HUVEC tube formation and migration triggered by TAZ overexpression in H460 cells. But this inhibition was also counteracted by the up-regulation of AREG in A549 cells (Fig. 6a–d). We measured the protein expression levels of components in EGFR signaling pathway and endothelial markers in these co-transfected cells. Silencing AREG suppressed the phosphorylation of Akt, ERK1/2, and EGFR in H460 cells. Moreover, the suppressed levels of phospho-Akt, phospho-ERK1/2 and phospho-EGFR resulting from the knockdown of TAZ were raised up after AREG overexpression in A549 cells (Fig. 6e). For endothelial markers, downregulation of AREG inhibited the expression of PECAM-1 and PROX-1 in H460 cells. And upregulation of AREG increased the expression of PECAM-1 and PROX-1 in A549 cells. Taken together, the tumorigenic ability enhanced by TAZ was mediated by AREG. Additionally, AREG activated the EGFR signaling pathway in EGFR wild-type NSCLC cell lines.

### TAZ promoted tumor growth and sensitized EGFR wild-type NSCLC to gefitinib by targeting AREG in vivo

Xenografts were generated by H460 cells that had been co-transfected with pEX2-TAZ and shNC or pEX2-TAZ and shAREG. As shown in Fig. 6f, g, downregulation of AREG slowed down the tumor growth caused by overexpression of TAZ. Moreover, after the treatment of gefitinib, the mice injected with H460 pEX2-TAZ-shNC cells grew markedly smaller tumors than those injected with H460 pEX2-TAZ-shAREG cells (Fig. 6h, i).

## Discussion

Newly invented diagnostic technologies^19^ have revolutionized the treatment strategy for lung cancer, but most of the patients still face a poor prognosis until EFR-TKIs emerged^20^. Large clinical trials and studies have verified the efficacy of EGFR-TKIs in advanced NSCLC patients harboring EGFR-sensitive mutations^21–23^. Moreover, the clinical benefit of EGFR-TKIs sparks the discovery of other biomarkers or drugs that can predict or enhance the sensitivity of NSCLC to EGFR-TKIs in wild-type patients^24^.

**Fig. 7:**
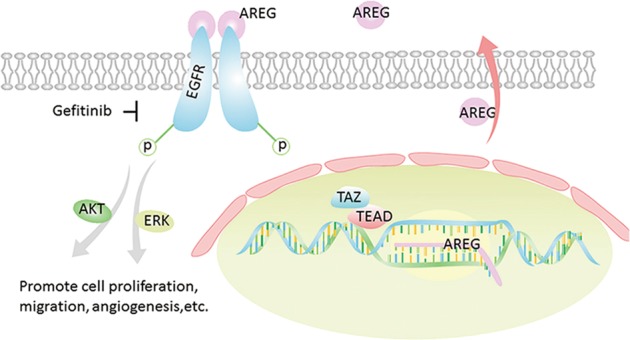
The mechanism through which TAZ activates EGFR signaling pathway and enhances the sensitivity of EGFR wild-type NSCLC to gefitinib

In EGFR variants associated with NSCLC, the receptor remains active in a ligand-independent manner^16^. We supposed that a ligand-dependent mechanism may regulate the activation of the receptor in EGFR wild-type NSCLC. As demonstrated in Fig. 7, TAZ interacted with TEAD, its main transcription factor, and induced the transcription of AREG. We assume that AREG, as the ligand of EGFR, may bind to EGFR and continuously activates the signaling pathway. As a consequence, the tumorigenesis is accelerated and gefitinib gets more powerful.

Our in vitro and in vivo experiments validated the assumption above. The role of TAZ in the proliferation and migration of NSCLC has already determined by previous researches^25–28^^,^ To our knowledge, this is the first report to show that the angiogenesis in NSCLC can be enhanced by TAZ, which expands our knowledge about the oncogenic function of TAZ. And our dual-luciferase reporter assays and ChIP-qPCR assays explained this mechanism at the molecular level.

Our observation is consistent with other opinions that high level of AREG increased gefitinib sensitivity in EGFR wild-type cancer cell lines^16^. Here, we determined the upstream of AREG and further explained the underlying mechanism. Moreover, another clinical study revealed that EGFR wild-type NSCLC patients who were AREG-positive achieved a higher disease control rate than those AREG negative (50% versus 25%) after EGFR-TKI therapy^17^. Sette et al. found that Tyr1068-phosphorylated EGFR could be used as a biomarker to indicate a better response to erlotinib in EGFR wild-type lung cancer. This finding corroborates the results of our study in which the phosphorylation site of p-EGFR was just Tyr1068^29^.

However, a recent study showed that panobinostat inhibited the transcription of TAZ, and panobinostat combined with gefitinib was more effective than gefitinib alone^30^. This study advocated the benefit of TAZ knockdown and suggested TAZ as a biomarker for gefitinib resistance. It is a study conflicting ours. The reason may be that in this study, EGFR expression was reduced in the panobinostat-treated group rather than the gefitinib group, and panobinostat showed a stronger effect than gefitinib in driving cell apoptosis. Hence, panobinostat played a leading role in tumor suppression. Besides, since TAZ is an oncogene, the inhibition of TAZ suppressed the tumor growth in their study.

In our study, we focused on the relationship between the baseline TAZ level and gefitinib sensitivity. Relative higher cell viability in the TAZ low expression group after gefitinib treatment showed the antitumor effect was caused by gefitinib but not TAZ knockdown. We should accept that oncogenes can sometimes be beneficial in cancer therapy. As Xie et al. found in a clinical trial, NSCLC patients with high TAZ expression obtained a pronounced better survival after adjuvant chemotherapy than those with low TAZ expression^31^.

Although some in vivo experiments indicated TAZ could not serve as a driver gene in NSCLC^14,15^, TAZ still exerts profound impact on lung cancer development^32^. Our team had previously confirmed that TAZ changed gefitinib sensitivity in the presence of EGFR-T790M mutation in lung adenocarcinoma^33^. This suggests that TAZ and gefitinib may interact in the lung cancers of different gene types. To think more, except for the receptor mutation, the increased gene copy number of receptor^8^, and the increased expression of the ligand as is mentioned in this study, does the mutation of the ligand also relate to the EGFR signaling and the EGFR-TKIs? In addition, homologous to TAZ, YAP(yes-associated protein) displays a similar organization in 46% of the amino acid sequences that have been identified^10^. However, both types of genes function in different ways. For example, the YAP-deficient mouse embryos showed severe developmental perturbation which could not be compensated by TAZ^34^. We will continue to testify the association between YAP and EGFR-TKIs sensitivity, and the potential use of TAZ in the development of anti-angiogenesis drugs.

However, the limitation also lies. The finding in this animal model should be tested in a patient population large enough. And whether other genes participate in the enhanced gefitinib sensitivity deserves further investigation.

In conclusion, TAZ sensitized EGFR wild-type NSCLC to gefitinib. TAZ promoted the transcription of AREG via TEAD, thereby activating EGFR signaling pathway, and ultimately arousing tumorigenesis and drug sensitivity. Our study has revealed that TAZ is not only a potential therapeutic target but also an adjunct biomarker for EGFR genotype if EGFR-TKIs are used for NSCLC patients.

## Data Availability

All data in our study are available upon request.
